# Conditional overexpression of TGFβ1 promotes pulmonary inflammation, apoptosis and mortality via TGFβR2 in the developing mouse lung

**DOI:** 10.1186/s12931-014-0162-6

**Published:** 2015-01-16

**Authors:** Angara Sureshbabu, Mansoor A Syed, Chandra Sekhar Boddupalli, Madhav V Dhodapkar, Robert J Homer, Parviz Minoo, Vineet Bhandari

**Affiliations:** Department of Pediatrics, Yale University School of Medicine, 333 Cedar Street, New Haven, CT 06510 USA; Department of Medicine and Yale Cancer Center, Yale University School of Medicine, 333 Cedar Street, New Haven, CT 06510 USA; Department of Pathology, Yale University School of Medicine, 333 Cedar Street, New Haven, CT 06520 USA; Department of Pediatrics, University of Southern California, 1200 North State Street, Los Angeles, CA 90033 USA

**Keywords:** Transforming growth factor, Oxygen, Inflammation, Cell death, Angiopoietin, Newborn, Pulmonary, Bronchopulmonary dysplasia

## Abstract

**Background:**

Earlier studies have reported that transforming growth factor beta 1(TGFβ1) is a critical mediator of hyperoxia-induced acute lung injury (HALI) in developing lungs, leading to impaired alveolarization and a pulmonary phenotype of bronchopulmonary dysplasia (BPD). However, the mechanisms responsible for the TGFβ1-induced inflammatory signals that lead to cell death and abnormal alveolarization are poorly understood. We hypothesized that TGFβ1 signaling via TGFβR2 is necessary for the pathogenesis of the BPD pulmonary phenotype resulting from HALI.

**Methods:**

We utilized lung epithelial cell-specific TGFβ1 overexpressing transgenic and TGFβR2 null mutant mice to evaluate the effects on neonatal mortality as well as pulmonary inflammation and apoptosis in developing lungs. Lung morphometry was performed to determine the impaired alveolarization and multicolor flow cytometry studies were performed to detect inflammatory macrophages and monocytes in lungs. Apoptotic cell death was measured with TUNEL assay, immunohistochemistry and western blotting and protein expression of angiogenic mediators were also analyzed.

**Results:**

Our data reveals that increased TGFβ1 expression in newborn mice lungs leads to increased mortality, macrophage and immature monocyte infiltration, apoptotic cell death specifically in Type II alveolar epithelial cells (AECs), impaired alveolarization, and dysregulated angiogenic molecular markers.

**Conclusions:**

Our study has demonstrated the potential role of inhibition of TGFβ1 signaling via TGFβR2 for improved survival, reduced inflammation and apoptosis that may provide insights for the development of potential therapeutic strategies targeted against HALI and BPD.

## Background

Transforming growth factor beta 1 (TGFβ1) is a secretory cytokine that binds to the Type II TGFβ receptor (TGFβR2), which then complexes with the type I TGFβ receptor (TGFβRI or ALK1 or ALK5). This binding initiates TGFβ signaling via smad phosphorylation and nuclear translocation [1]. There is growing evidence that TGFβ signaling is involved in the regulation of branching and septation phases of lung development [2,3]. In contrast, TGFβ1 has been documented to induce inflammation and apoptosis in lung epithelial cells [4,5].

Premature infants require supplemental oxygen and/or mechanical ventilation for prolonged time periods, and may subsequently develop Bronchopulmonary Dysplasia (BPD). BPD, the most common chronic respiratory disease in infants, is characterized by the presence of impaired alveolarization and dysregulated vascularization [6]. Understanding the mechanisms responsible for the development of BPD has focused on the identification of signaling or remodeling molecules that are crucial in lung development and/or response to lung injury. One such signaling pathway that is known to be a central mediator in hyperoxia induced acute lung injury (HALI) and BPD is TGFβ signaling [1,7]. Previous studies have demonstrated increased expression of TGFβ, both total and bioactive forms, in bronchoalveolar lavage fluid and tracheal aspirates of infants developing BPD [8,9].

Many studies have demonstrated that TGFβ1 is a critical regulator of HALI [10,11]. Hyperoxia mediated TGFβ1 sets into flow a deluge of inflammation [12,13]. However, the mechanisms responsible for the TGFβ1-induced immunogenicity and the levels of inflammatory signals that lead to cell death are poorly understood. Therefore, this study was aimed to investigate the quantitation of endogenous immune cells regulated by TGFβ1, which result in cell death. In addition, we wanted to assess the role of TGFβ1 in alveolarization and impact on molecular mediators known to be associated with development of HALI and BPD. Furthermore, we wanted to evaluate if all or some of the above-mentioned effects of TGFβ1 signaling were mediated via TGFβR2.

We hypothesized that TGFβ1 signaling via TGFβR2 is necessary for the pathogenesis of HALI and the BPD phenotype resulting from hyperoxia. Therefore, we used lung epithelial cell-specific TGFβ1 transgenic (TG) mice model to demonstrate TGFβ1-induced inflammation and apoptosis in developing lungs. In addition, we also evaluated Angiopoietin (ANGPT) 1 and ANGPT2 protein expression in the above model systems as ANGPT2 has been described to have a critical role in HALI and BPD [14,15]. Furthermore, in order to assess the receptor specificity of these effects, we used lung epithelial cell-specific TGFβR2 null mutant (knockout or KO) mice to evaluate the effects of TGFβ1-induction on mortality as well as pulmonary inflammation and apoptosis in developing lungs.

## Methods

### Animals

All animal studies were approved by the Institutional Animal Care and Use Committee at the Yale University School of Medicine. The TGFβ1TG mice were generated as previously described [3,16], and express TGFβ1 with maternal exposure to doxycycline (dox) in the drinking water. These triple-TGFβ1 TG mice are expressing the active form of human TGFβ1 and the levels and downstream effects of the same (upon dox activation) over time have been previously reported [16]. Maternal exposure to dox was performed from postnatal (PN) day 1–7 or from PN7 to PN10 [17]. Lung epithelial-cell specific deletion of TGFβR2 (TGFβR2KO) has been recently described and these mice were utilized for our experiments [18].

### Bronchoalveolar lavage fluid collection

The mouse lungs were lavaged three times with 300 microliters of PBS using standard techniques and inflation pressure, as previously described [19]. The bronchoalveolar lavage fluid (BALF) was centrifuged at 400 g for 5 min at 4°C. The cell pellet was re-suspended in 1% FBS for FACS analysis.

### Tissue preparation

Mouse pups were sacrificed at PN7 or PN10. They were subjected to a standard protocol for lung inflation (25 cm) [20]. The right lungs were immediately flash frozen using liquid nitrogen and immediately stored at −80°C while the left lungs were fixed in 10% neutral buffered formalin and processed for histological analysis.

### Oxygen exposure

For the exposure to hyperoxia (100% O_2_), newborn (NB) mice (along with their mothers) were placed in cages in an airtight Plexiglas chamber (55 × 40 × 50 cm), as described previously [21-23]. Exposure to oxygen was initiated on PN1 and continued till PN7 of life. Two lactating dams were used. Mothers were alternated in hyperoxia and room air (RA) every 24 h. The litter size was kept limited to 12 pups to control for the effects of litter size on nutrition and growth. Throughout the experiment, they were given free access to food and water. Oxygen levels were constantly monitored by an oxygen sensor that was connected to a relay switch incorporated into the oxygen supply circuit. The inside of the chamber was kept at atmospheric pressure, and mice were exposed to a 12 h light–dark cycle.

### Lung morphometry

H&E stained sections were photographed using an Olympus microscope with an in built digital camera system at 100× magnification (Olympus, Tokyo, Japan). Alveolar size was estimated from the mean chord length of the airspace, as previously described [19]. Alveolar septal wall thickness was estimated using Image J software, adapting the method described previously for bone trabecular thickness, for the lung [19]. 10× magnification images were obtained with Olympus 1X70 Microscope using Cellsens Dimension software. Radial alveolar count (RAC) and secondary septal crests were done as previously described [24,25].

### TUNEL assay

For apoptosis quantification, TdT-mediated dUTP nick end labeling (TUNEL) staining was performed according to manufacturer’s instructions keeping positive and negative controls (Roche Diagnostics). 20× magnification images were obtained with Olympus 1X70 Microscope using Cellsens Dimension software. Briefly 5 areas were selected (covers almost the entire histologic specimen) from each slide and 200 cells were counted using Cellsens software from Olympus microscope, followed by manually counting of TUNEL positive cells. The TUNEL index was calculated by randomly selecting 3 of the 5 high-power fields in each slide, counting 200 cells in each area, and expressing the number of TUNEL-positive cells as a percentage [17].

### Immunohistochemistry

Whole lungs were isolated from NB PN7 and PN10 mice and immediately fixed in 10% neutral buffered saline. Lung serial sections were stained with antibodies prosurfactant protein C (Catalog #AB3786, Millipore), vWF (DAKO), Myeloperoxidase (Biovision) and cleaved caspase 3 (Catalog #9661, Cell Signaling Technology). Sections were developed with Vectastain ABC kit (Catalog #PK4001, Vector laboratories) followed by hematoxylin staining (Catalog #H3401, Vector laboratories). 10× or 20× magnification images were obtained with Olympus 1X70 Microscope using Cellsens Dimension software and vWF staining was quantified by image J software.

### Myeloperoxidase (MPO) ELISA from NB lung tissue

The snap-frozen lungs were thawed, weighed, and transferred to different tubes on ice containing 1 ml of Tissue Protein Extraction Reagent (T-PER; Pierce Biotechnology). Cells or lung tissues were homogenized at 4°C. Cell or lung homogenates were centrifuged at 10,000 g for 10 min at 4°C. Supernatants were transferred to clean microcentrifuge tubes. Total protein concentrations in the lung tissue homogenates were determined using a Biorad protein assay kit (Catalog #500–0006; Biorad) and MPO levels were evaluated in lung tissue homogenates using a mouse ELISA kit (Catalog #ab155458; Abcam) according to the user’s manual.

### Western blot analysis

NB mice whole lungs were harvested in lysis buffer and protein concentrations were determined using Bradford assay [19]. Protein extracts (both cellular and tissue homogenates) were loaded on to a Mini-Protean TGX gel electrophoretic system. Proteins were electroblotted onto an immunoblot PVDF membrane (Biorad catalog #162–0177). After transfer, non-specific binding was prevented with 5% skimmed milk TBS-Tween. After blocking, the membrane was incubated with anti-phospho AKT (ser 473) (Catalog #4060), anti-caspase-3 (Catalog #9662), and anti-cleaved caspase 3 (Catalog #9662) obtained from Cell Signaling Technology, USA. Anti-ANGPT1 (Catalog #AB10516), anti-ANGPT2 (Catalog #176002) and anti-Endoglin (Catalog #05-1424) were obtained from Millipore, USA. Chemiluminescent bands were visualized using SuperSignal West Pico or Femto substrate (Thermo Scientific, Rockford, IL). Rabbit polyclonal or monoclonal antibodies reactive to above proteins of interest were purchased from Cell Signaling Technology, Inc. (Danvers, MA). Quantification of western blots was performed using Image J software version 1.46 (NIH, Bethesda, MD).

### Multicolor flow cytometry

After the indicated duration of dox exposure to NB TGFβ1TG and TGFβ1TG X TGFβR2KO mice, they were sacrificed and their lungs were perfused through the right ventricle with 5 ml of ice cold PBS. The lungs were excised and single cell suspensions were prepared using digestion buffer as previously described [26]. Resultant single cell suspensions were stained for respective surface antigens and subsequently acquired on BD-LSRII flow cytometer. FACS analysis was performed with the use of FlowJo software.

### Antibodies

The following monoclonal antibodies directed to the respective proteins, conjugated to different flurochromes were used. Ly6c (clone: HK1.4), F4/80 (clone: BM8), CD45 (clone: 30-F11), CD103 (clone: 2E7) and MHC-II (clone: M5/114.152) were purchased from eBioscience (San Diego, CA). CD11c (clone: HL3) and CD11b (clone: M1/70) were purchased from BD Biosciences (San Jose, CA). CD206 (clone: C068C2) was purchased from BioLegend (San Diego, CA). All the antibodies that were used for FACS were monoclonals and highly specific. These were the concentrations of antibodies: CD11c (1 μg/ml), CD11b (0.05 μg/ml), F4/80 (0.2 μg/ml), CD206 (0.2 μg/ml), Ly6c (0.05 μg/ml), MHC-II (0.05 μg/ml).

### Statistical analysis

All statistical analyses were performed using Graph Pad Prism, version 5.0 (GraphPad Software, San Diego CA). The data were expressed as the mean ± SEM of a minimum of 3 independent experiments with a minimum of 4 mice in each group. Groups were compared with the Student’s two-tailed unpaired t test, 1-way ANOVA (followed by Tukey’s Multiple Comparison post-hoc test) or the Kaplan Meier Survival Analysis, as appropriate, using GraphPad Prism 5.0. A ‘p’ value of < 0.05 was considered statistically significant in all tests.

## Results

### Effect on survival, in room air, of lung epithelial cell-specific conditionally overexpressing TGFβ1TG mice lacking lung epithelial-cell specific TGFβR2

**Figure 1:**
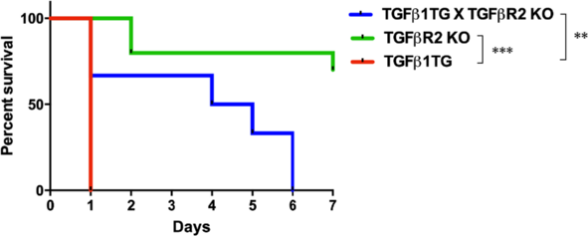
**Effect of TGFβ1 exposure on overall survival in maternally exposed doxycycline to NB TGFβTG, and TGFβTG X TGFβR2KO mice.** NB TGFβ1TG, and TGFβ1TG X TGFβR2KO littermate mice were exposed to maternal dox at PN1 - PN7 and then overall survival was assessed. Where NB: newborn; PN: postnatal; TGFβ1TG: TGFβ1 transgenic mice; TGFβ1TG X TGFβR2KO: TGFβ1 transgene mice crossed with TGFβ receptor II knockout mice. N = 7 in each group; **p < 0.01 and ***p < 0.001 TGFβ1TG vs. TGFβ1TG X TGFβR2KO and TGFβR2KO vs. TGFβ1TG respectively.

Since NB TGFβ1TG mice are known to have a high mortality upon dox activation in room air, in the first PN week, as previously reported [3], we evaluated the role of TGFβ1-signaling via TGFβR2 in lung epithelial cells *in vivo* on survival. Hence, we assessed survival in TGFβ1TG mice lacking TGFβR2, upon TGFβ1 activation from PN1 – PN7. TGFβ1TG X TGFβR2KO mice showed significantly lower mortality as compared to lung targeted overexpressing TGFβ1TG mice (Figure 1).

This suggests that lung-mediated signaling via TGFβR2 has such a significant effect that it can overcome the dramatic mortality that has been observed when increased concentrations of TGFβ1 are present at these critical (saccular/early alveolar) stages of lung development.

### Characterization of pulmonary (lung tissue and BALF) inflammatory myeloid compartment, in room air, of lung epithelial cell-specific conditionally overexpressing TGFβ1TG mice lacking lung epithelial-cell specific TGFβR2

It was initially reported that TGFβ1 overexpression in mice lungs leads to mononuclear-rich inflammation [16]. Since our aim in the present manuscript was to understand TGFβ1-mediated immune signaling in hyperoxia-induced cell death in neonates, we decided to focus on evaluating the macrophage/monocyte populations in the lung by flow cytometry. We hypothesized that reduced inflammation in lung tissue myeloid compartment could lead to TGFβ1TG X TGFβR2KO mice survival, and hence we evaluated this response. The mononuclear phagocytic system (monocytes, macrophages and dendritic cells) collectively plays a critical role in the maintenance of tissue integrity [27]. To evaluate the role of the mononuclear phagocytic system, TGFβ1TG, WT and TGFβTG X TGFβR2KO mice lungs and BALF cells were phenotypically analyzed for pro-inflammatory macrophages and lung tissue resident macrophages. Monocytes were analyzed for Ly6C^high^ and Ly6C^low^ cells. Lung pro-inflammatory macrophages resemble M1-like macrophages and are CD45^+^ F4/80^+^ CD206^−^ and CD11c^−^, whereas tissue resident macrophages resemble the M2 phenotype, expressing CD45^+^ F4/80^+^ CD206^+^ and CD11c^+^.

**Figure 2:**
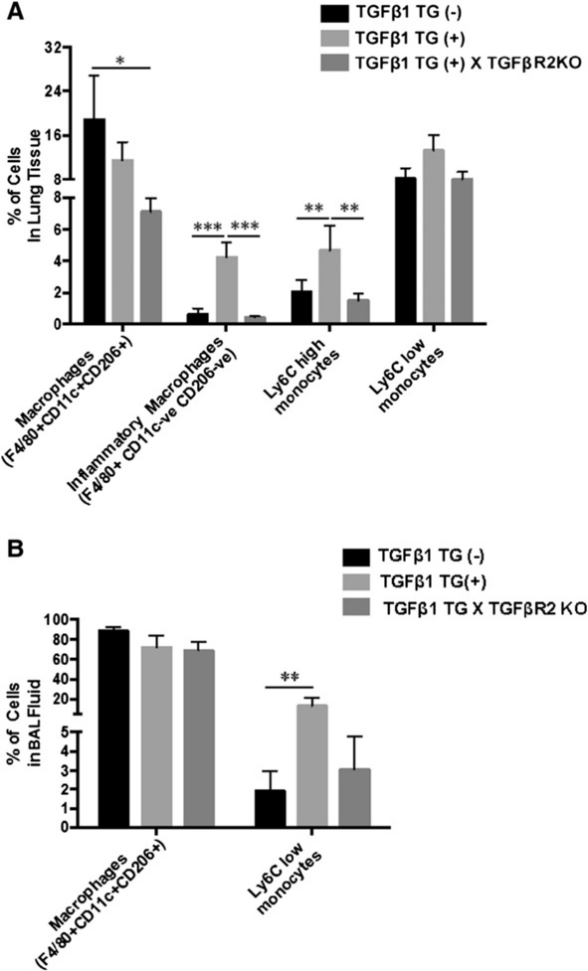
**TGFβ1-induced inflammation in TGFβ1TG and TGFβ1TG X TGFβR2KO mice.** Multicolor flow cytometric analysis showed significantly greater staining of inflammatory macrophages and LY6C high monocytes in TGFβ1TG mice lungs as compared to WT controls. TGFβ1TG X TGFβR2KO developing lungs showed significantly decreased staining of inflammatory macrophages and LY6C high monocytes as compared to TGFβ1TG mice. **(B)** Ly6Clow monocytes were significantly decreased in the BALF of WT mice, as compared with TGFβ1TG mice. BALF: bronchoalveolar lavage fluid. N = 4 in each group; *p < 0.05, **p < 0.01 and ***p < 0.001.

We found significant reduction in inflammatory macrophages and Ly6C^high^ monocytes in the lung tissue of the TGFβ1TG X TGFβR2KO mice, compared to TGFβ1TG mice. No such differences in inflammatory macrophages and Ly6C^high^ monocytes were observed when compared to WT littermate lungs. The numbers of tissue resident macrophages were slightly reduced in the lungs of TGFβ1TG X TGFβR2KO mice when compared to WT littermates. We observed no differences in Ly6C^low^ monocytes among the groups (Figure 2A). Ly6C^low^ monocytes were significantly decreased in the BALF of TGFβ1TG X TGFβR2KO and WT mice, as compared with TGFβ1TG mice (Figure 2B).

Taken together, the above data suggests that signaling via TGFβR2, at least in part, mediates the pulmonary inflammatory response of TGFβ1 in developing lungs.

### Effect on lung apoptotic cell death specifically in Type II alveolar epithelial cells (AECs), in room air, of lung epithelial cell-specific conditionally overexpressing TGFβ1TG mice lacking lung epithelial-cell specific TGFβR2

**Figure 3:**
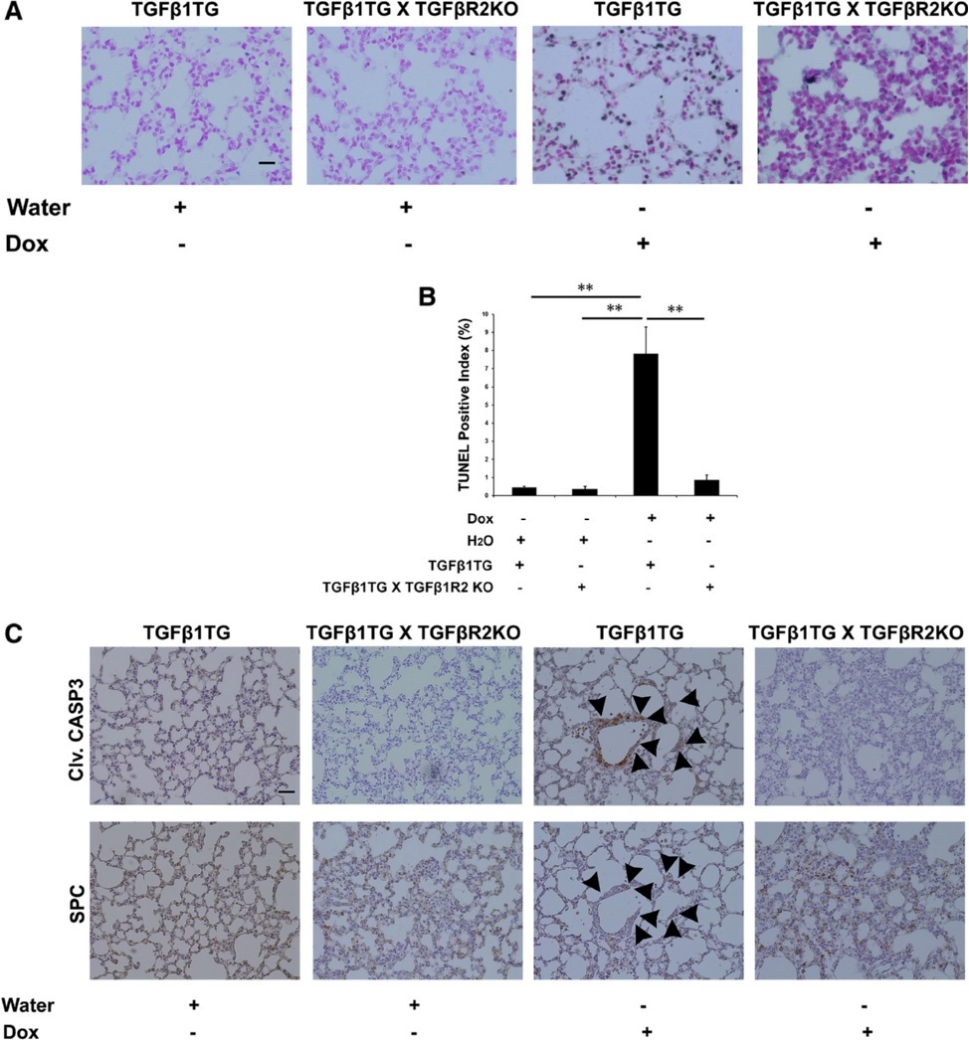
**Effect of TGFβ1 on lung apoptotic cell death. (A)** TdT-mediated dUTP nick end labeling (TUNEL) was assessed in NB TGFβ1TG and TGFβ1TG X TGFβR2KO littermates where maternal dox exposure from PN7 – PN10 was carried out. Scale bar: 50 μm **(B)** Bar graph showing the percentage of TUNEL positive cells indicating the apoptosis quantification in TGFβ1TG mice and TGFβ1TG X TGFβR2KO littermates. **(C)** Representative serial sectioning images of cleaved caspase 3 and surfactant protein C staining in the lung specimens of TGFβ1TG mice and TGFβ1TG X TGFβR2KO littermates. Scale bar: 50 μm.

Next, we evaluated cell death in lung targeted overexpressing TGFβ1TG mice and TGFβ1TG X TGFβR2KO mice from PN7 – PN10. We used TUNEL and immunohistochemistry respectively. TGFβ1TG X TGFβR2KO mice showed significantly decreased TUNEL positive index and decreased cleaved caspase 3 staining in Type II AECs (surfactant protein C positive), as compared to TGFβ1TG mice (Figure 3A – C).

### Effect on lung morphometry and vessel density, in room air, of lung epithelial cell-specific conditionally overexpressing TGFβ1TG mice lacking lung epithelial-cell specific TGFβR2

**Figure 4:**
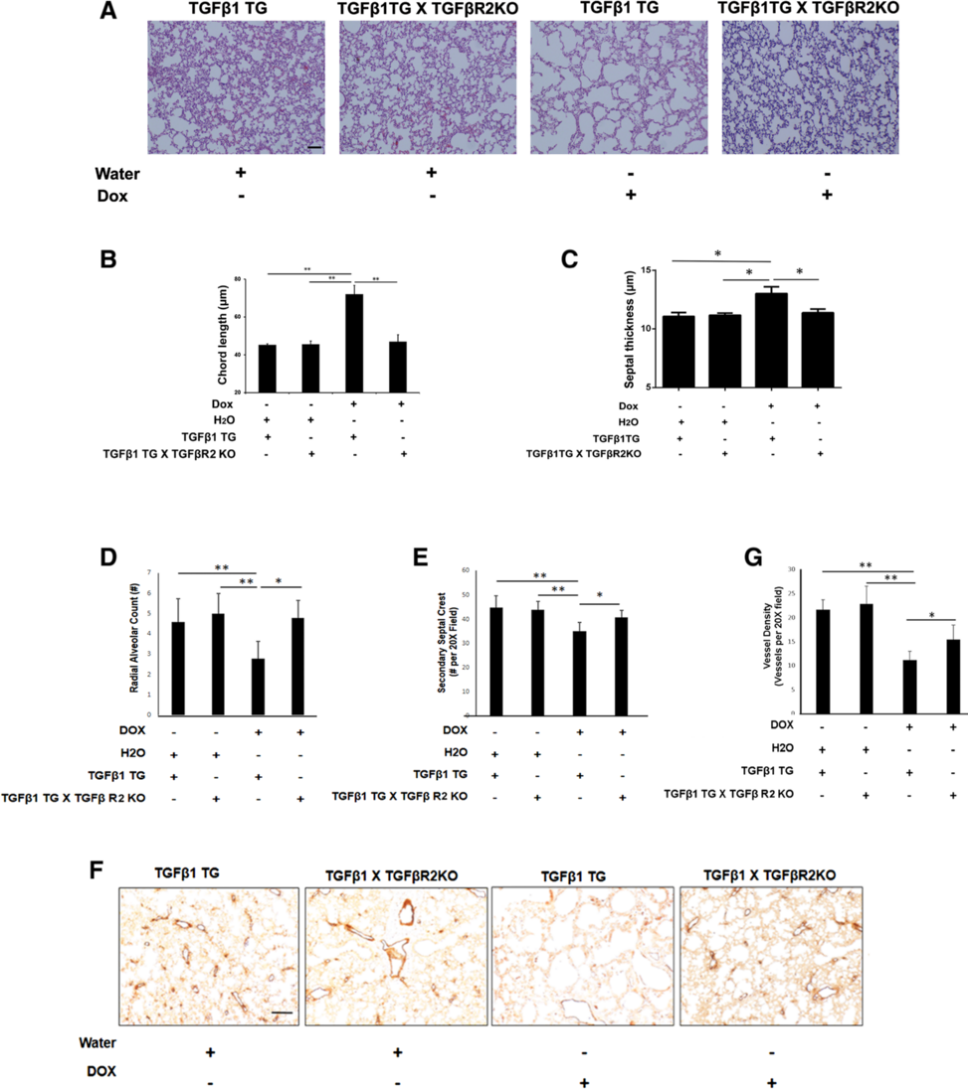
**Effect of TGFβ1 on lung morphometry. (A)** Representative images of lung histology (H&E stain) of NB TGFβ1TG mice and TGFβ1TG X TGFβR2KO mice. Scale bar: 100 μm **(B)** Bar graph showing the mean chord length in TGFβ1TG mice and TGFβ1TG X TGFβR2KO mice. **(C)** Bar graph showing the septal thickness in TGFβ1TG mice and TGFβ1TG X TGFβR2KO mice. **(D)** Bar graph showing the radial alveolar count in TGFβ1TG mice and TGFβ1TG X TGFβR2KO mice. **(E)** Bar graph showing the secondary septal crest numbers in TGFβ1TG mice and TGFβ1TG X TGFβR2KO mice. **(F)** Representative images of lung immunohistochemistry of vessel density (vWF staining) of NB TGFβ1TG mice and TGFβ1TG X TGFβR2KO mice. Scale bar: 100 μm **(G)** Bar graph showing quantification of vessel density in TGFβ1TG mice and TGFβ1TG X TGFβR2KO mice. N = 4, in each group; *p < 0.05, **p < 0.01.

Lung architecture was assessed and chord length as well as septal thickness were found to be significantly decreased in TGFβ1TG X TGFβR2KO as compared to TGFβ1TG mice (Figure 4A – C). In concordance, we found the RAC and secondary septal crest numbers as well as vessel density improved in the TGFβ1TG X TGFβR2KO as compared to TGFβ1TG mice (Figure 4D – G). This suggested that absence of TGFβR2 in TGFβ1 signaling resulted in decreased apoptotic cell death and preserved alveolar architecture as well as vascular development.

### Effect on molecular mediators, in room air, of lung epithelial cell-specific conditionally overexpressing TGFβ1TG mice lacking lung epithelial-cell specific TGFβR2

**Figure 5:**
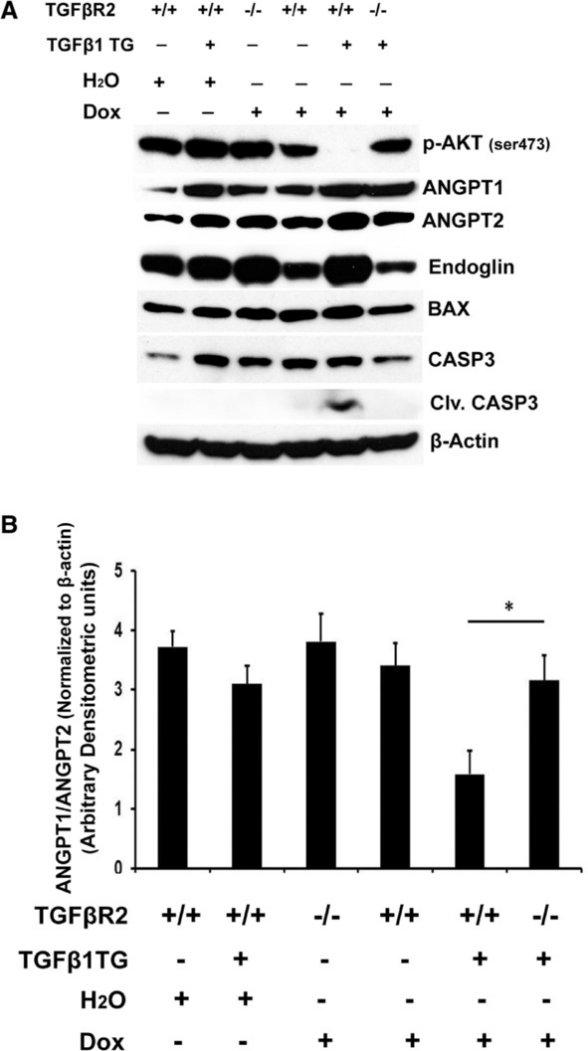
**TGFβ1 signaling in NB TGFβ1TG, and TGFβ1TG X TGFβR2KO mice lungs. (A)** Western blot analysis showed upregulation of phospho AKT, and down regulation of ANGPT2, and pro-apoptotic proteins (BAX; cleaved caspase 3) in NB TGFβ1TG X TGFβR2KO lungs as compared to TGFβ1TG mice. **(B)** Densitometric analysis showed increased ANGPT1 to ANGPT2 ratio in maternal dox exposed NB TGFβ1TG X TGFβR2KO lungs as compared to TGFβTG mice. N = 3, in each group; *p < 0.05.

To further understand the mechanism of TGFβ1-induced cell death, we examined molecular markers including endoglin (or TGFβR3), ANGPT1, ANGPT2, Phospho-AKT (Ser 473), BAX and cleaved caspase 3. We noted that Phospho-AKT was increased while endoglin, BAX, caspase 3 and cleaved caspase 3 were decreased in TGFβ1TG X TGFβR2KO as compared to TGFβ1TG mice lungs (Figure 5A). Densitometric analysis showed significantly increased ANGPT1 to ANGPT2 ratio in TGFβ1 exposed NB TGFβTG X TGFβR2KO lungs as compared to TGFβ1TG mice lungs (Figure 5B).

This suggested that the decreased apoptotic cell death response could be due to increased presence of molecular mediators of cell survival in the absence of TGFβR2 in TGFβ1 signaling processes in developing lungs.

### Effect on lung architecture, upon hyperoxia exposure, in TGFβR2KO mice

**Figure 6:**
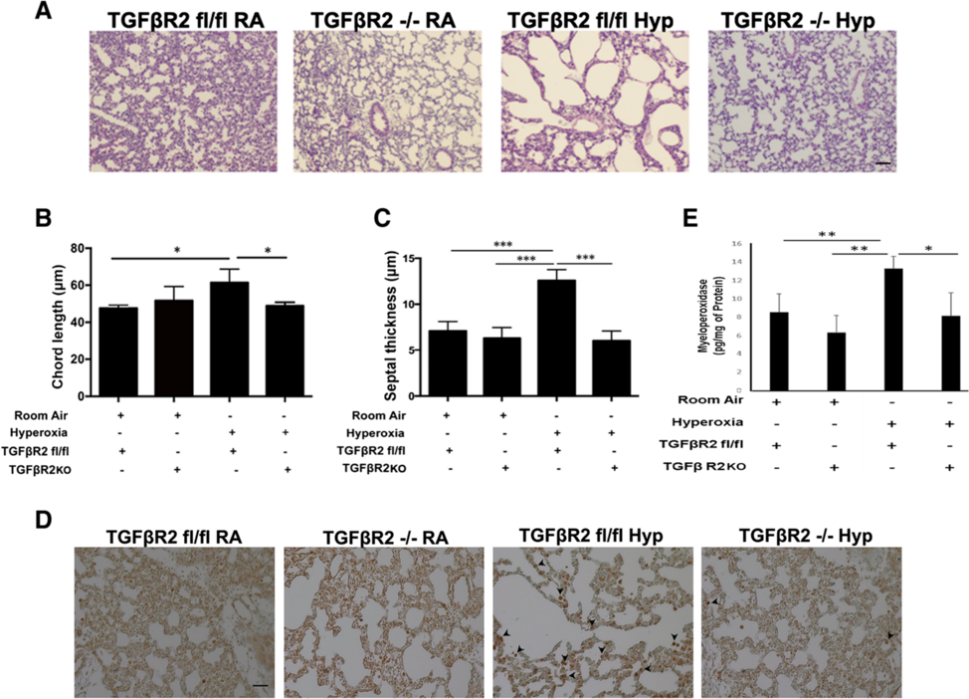
**TGFβR2KO mice have improved lung morphometry in hyperoxia settings. (A)** Representative images of lung histology (H&E stain) of NB TGFβR2KO mice exposed to RA or 100% O_2_ from PN1 – PN7 with appropriate controls. Scale bar: 100 μm **(B)** Bar graph showing morphometric analysis of mean chord length in hyperoxia exposed PN7 TGFβR2KO mice as compared to room air controls. **(C)** Bar graph showing morphometric analysis of septal thickness in hyperoxia exposed PN7 TGFβR2KO mice as compared to room air controls. **(D)** Representative images of immunohistochemistry of myeloperoxidase (MPO) staining showing of inflammatory cells (neutrophils) in the lungs NB TGFβR2KO mice exposed to RA or 100% O_2_ from PN1 – PN7 with appropriate controls. Scale bar: 100 μm **(E)** Bar graph showing MPO levels (measured by ELISA) in hyperoxia exposed PN7 TGFβR2KO mice as compared to room air controls. N = 4 in each group; *p < 0.05, ***p < 0.001.

Given the fact that hyperoxia exposure is known to increase TGFβ in developing lungs and lead to abnormal alveolarization [1,11,13,28,29], we finally examined if lack of signaling via its putative receptor TGFβR2, upon hyperoxia exposure, would have an impact on the pulmonary phenotype. Hence, we assessed the lung architecture in TGFβR2KO mice exposed to hyperoxia from PN1 – PN7 and confirmed our histologic observations by chord length and septal thickness measurements. Chord length and septal thickness were significantly decreased in hyperoxia exposed TGFβR2KO mice as compared to TGFβR2 flox (control) mice (Figure 6A - C). In addition, we noted a significant decrease in the homing of inflammatory cells (neutrophils), as detected by MPO staining (Figure 6D - E).

This protective effect in the hyperoxia-exposed TGFβR2KO mice lungs was further confirmation of our results of TGFβ1-induced signaling being mediated through this receptor having a significant impact on TGFβ1-mediated effects in developing lungs.

**Figure 7:**
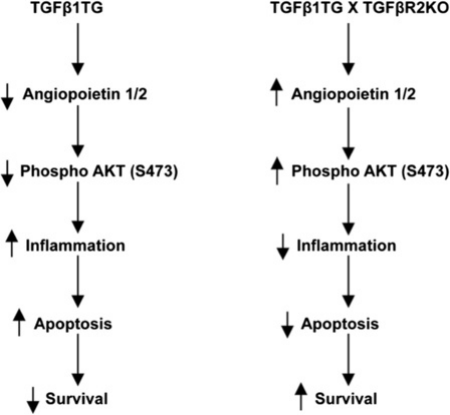
**Proposed schema of the mechanism of TGFβ1-induced TGFβR2-mediated effects in the developing lung.** A proposed model illustrating the mechanism of TGFβ1-induced specific molecular mediators resulting in decreased inflammation, apoptosis and mortality in NB TGFβ1TG X TGFβR2KO, as compared to TGFβ1TG mice.

To summarize, we show that the detrimental effects of increased mortality, pulmonary inflammation, cell death and impaired alveolarization of enhanced lung epithelial cell specific TGFβ1 signaling in developing lungs are mediated, in part, via TGFβR2. In addition, specific molecular mediators of cell survival are associated with these responses in developing lungs. A proposed model of these molecular mediators leading to their ultimate effects has been shown in Figure 7.

## Discussion

In the present study, lung epithelial cell specific targeted conditional TGFβ1TG and epithelial cell specific TGFβR2 conditional null mutant mice were used to explore the molecular mechanisms of TGFβ1-induced injury in developing lungs. This context is particularly important for the study of HALI and BPD that arise in the critical developing stages of lungs, with abnormal TGFβ1 signaling *in vivo* [30]. Although the pathogenesis of BPD is not well understood, increased TGFβ (secondary to hyperoxia exposure) signaling in the saccular/early alveolar stages is said to be one of the causative factors for the development of impaired alveolarization secondary to inflammation [1,31,32].

High levels of TGFβ1 (as well as β2 and β3) immunoreactivity have been reported in the developing lung in the bronchial and alveolar epithelium of the NB mouse [13]. Furthermore, hyperoxia exposure increases TGFβ-signaling [13]. A TGFβ-neutralizing antibody (1D11) was effective in improving alveolarization, extracellular cellular matrix assembly and microvascular development in NB mice exposed to hyperoxia (85% O_2_) for 10 PN days [13]. However, 1D11 is a pan-specific TGFβ IgG1 antibody that targets all the 3 isoforms of TGFβ i.e. 1–3 [13]. In our earlier report, exposure of hyperoxia (100% O_2_) to the lung epithelial cell targeted TGFβ1TG mice led to increased mortality, which was significantly diminished by using a c-JunNH2-terminal kinase (JNK) pathway inhibitor [17]. In addition, the JNK-pathway inhibitor was able to ameliorate the BPD pulmonary phenotype of impaired alveolarization in the TGFβ1TG mice [17]. Chronic hyperoxia (85% O_2_) exposure from PN1 to PN28 in mice led to a 4-fold increase of TGFβR2 (among other TGFβ signaling molecules), along with impaired alveolarization characterized by approximately doubling of the mean linear intercept, in the lung [1].

Our work extends the above observations by providing specificity of the effects of the TGFβ1 isoform as well as its signaling via the specific receptor TGFβR2. In addition, by using lung epithelial cell targeted overexpression and null mutant mouse models, respectively, we are able to localize the effects to be secondary to the production of TGFβ1 and inhibition of its signaling in particular cell types in the developing lung.

As mentioned above, hyperoxia contributes significantly to the development of HALI and BPD in neonates [33]. To this end, we were able to demonstrate the specificity of such a protective response, as evidenced by TGFβ1TG X TGFβR2KO mice having improved survival (up to PN6) as compared to PN1 of TGFβ1TG mice, in room air. The fact that this survival advantage was noted in room air lends credence to our hypothesis that this is a specific TGFβ1-induced TGFβR2-mediated effect, and not confounded potentially by other mediators that are activated/released upon hyperoxia exposure.

Other investigators have reported that TGFβ1 induced inflammation in adult lung injury model systems [34], but we were unable to locate any such effects reported in NB mice. Adding novel information, we demonstrated diminished pulmonary inflammation in TGFβ1TG mice in which TGFβR2 signaling had been abrogated. In addition, we were able to demonstrate that specifically it was the accumulation of inflammatory macrophages and monocytes in the lungs of TGFβ1TG mice. In contrast, TGFβ1TG X TGFβR2KO NB mice were found to have diminished levels of pro-inflammatory macrophages.

We have reported earlier that hyperoxia exposure increases TGFβ1 expression as well as cell death in A549 cells upon hyperoxia exposure in a dose-dependent manner [17]. Others have shown that exposure to hyperoxia of primary alveolar epithelial Type II cells led to increased sensitivity to TGFβ-induced apoptosis [1]. Furthermore, we provide data that worsening of lung morphometric indices (chord length, septal thickness, RAC and secondary septal crest numbers) and decreased vessel density in the TGFβ1TG mice were dependent, in part, by signaling via TGFβR2. In support of these observations, we provide novel mechanistic *in vivo* data of increased apoptosis via the mitochondrial active BAX dependent pathway in the presence of increased TGFβ1 in developing lungs, which is abrogated by concomitant inhibition of TGFβR2 signaling.

We have previously reported that TGFβ1TG mice lungs have increased expression of apoptotic cell death mediators, specifically Fas-L and caspase-3 [17]. In the present study, we extend those observations by showing increased BAX and cleaved caspase-3.

We found increased ANGPT2, and Phospho-AKT (Ser 473), but no change in ANGPT1. We have previously reported on the role of increased ANGPT2 as a critical contributing factor in HALI in adult [14,35] and neonatal [19,23] mice. In addition, we have also noted a significant association of increased ANGPT2 in hyperoxia-induced human diseases in adults (ALI) [14] as well as neonates (BPD) [14,15]. Since TGFβ1 has been shown to be associated with BPD and overexpression lung models of TGFβ1 mimic the pulmonary phenotype of BPD as reported by us [17] and others [3,36], our data implicates ANGPT2 as a novel downstream molecule amenable to potential therapeutic modulation in HALI in the NB lung. Since ANGPT1 levels did not change and both ANGPT 1 and 2 signal through the Tie2 receptor, our data suggest that targeting the ligand ANGPT2 in HALI would be the preferred option in such a scenario. Endoglin is part of the TGFβR complex and has been shown to be increased in human BPD [37]. Our data would suggest that TGFβ1 signaling increases the expression of endoglin. AKT is a well-known survival factor reported to have a role in different experimental models of BPD [38-41]; we now implicate AKT as a down streaming signaling molecule in our TGFβ1 overexpressing mouse lung model of BPD. We further confirmed the specificity of the TGFβ1 signaling pathway by noting the reversal of the effects noted above in the TGFβ1TG X TGFβR2KO mice lungs.

Among other models of BPD, cyclical stretch during invasive mechanical ventilation in NB mice in room air and/or hyperoxia (40% O_2_) has also been reported to increase TGFβ-signaling and apoptotic cell death, including cleaved caspase 3 [42-44]. Activation of AKT has been shown to protect NB rats from HALI and BPD [39].

Investigators have reported additional molecules in NB mice lungs that have been associated with inhibition of TGFβ-signaling to improve the pulmonary phenotype relevant to HALI and BPD [11,45-47]. As also mentioned earlier, increased pulmonary levels of TGFβ have been reported in association with human BPD [9,48,49]. Interestingly, increased endoglin [37], decreased ANGPT1 [37], and increased ANGPT2 [14,15] in the NB lung have all been associated with human BPD.

To explore the wider importance, we finally subjected TGFβR2KO mice to hyperoxia exposure from PN1 – PN7. Lung architecture (as measured by chord length and septal thickness) was significantly decreased (with equivalency to room air controls), in addition to decreased inflammation, in TGFβR2KO hyperoxia-exposed mice lungs suggesting inhibition of TGFβ1 signaling mediated via its receptor TGFβR2 has a protective role in hyperoxia settings.

In conclusion, our data provides evidence suggesting TGFβR2KO comprehensively prevents the deleterious effects of TGFβ1 signaling by increased ANGPT1/ANGPT2 ratio, phosphorylation of AKT and decreased cleavage caspase 3 expression (including specifically in Type II AECs). Our study has demonstrated the potential role of inhibition of TGFβ1 signaling via TGFβR2 for improved survival, reduced inflammation and apoptosis that may provide insights for the development of novel therapeutic strategies targeted against HALI and BPD.
